# Sparse Project VCF: efficient encoding of population genotype matrices

**DOI:** 10.1093/bioinformatics/btaa1004

**Published:** 2020-12-10

**Authors:** Michael F Lin, Xiaodong Bai, William J Salerno, Jeffrey G Reid

**Affiliations:** btaa1004-aff1 mlin.net LLC, San Jose, CA 95113, USA; btaa1004-aff2 Department of Regeneron Pharmaceuticals, Inc., Regeneron Genetics Center, Tarrytown, NY 10591, USA

## Abstract

**Summary:**

Variant Call Format (VCF), the prevailing representation for germline genotypes in population sequencing, suffers rapid size growth as larger cohorts are sequenced and more rare variants are discovered. We present Sparse Project VCF (spVCF), an evolution of VCF with judicious entropy reduction and run-length encoding, delivering >10× size reduction for modern studies with practically minimal information loss. spVCF interoperates with VCF efficiently, including tabix-based random access. We demonstrate its effectiveness with the DiscovEHR and UK Biobank whole-exome sequencing cohorts.

**Availability and implementation:**

Apache-licensed reference implementation: github.com/mlin/spVCF.

**Supplementary information:**

Supplementary data are available at *Bioinformatics* online.

## 1 Introduction

Variant Call Format (VCF) is the prevailing representation for small germline variants discovered by high-throughput sequencing (Danecek *et al.*, 2011). In addition to capturing variants sequenced in one study participant, VCF can represent the genotypes for many participants at all discovered variant loci. This ‘Project VCF’ (pVCF) form is a 2-D matrix with loci down the rows and participants across the columns, filled in with each called genotype and annotations thereof, including quality-control (QC) measures like read depth, strand ratio and genotype likelihoods.

As the number of study participants *N* grows (columns), more variant loci are also discovered (rows), leading to super-linear growth of the pVCF genotype matrix. And, because cohort sequencing discovers mostly rare variants, this matrix consists largely of reference-identical genotypes and their high-entropy QC measures. In recent experiments with human whole-exome sequencing (WES), doubling *N* from 25 000 to 50 000 also increased the pVCF locus count by 43%, and 96% of all loci had non-reference allele frequency below 0.1% (Lin *et al.*, 2018). Empirically, vcf.gz file sizes in WES and whole-genome sequencing (WGS) are growing roughly with $N^{1.5}$ in the largest studies as of this writing (N≈500 000 WES). Unchecked, we project N=1 000 000 WGS will yield petabytes of *compressed* pVCF.

## 2 Approach

**Fig. 1.**
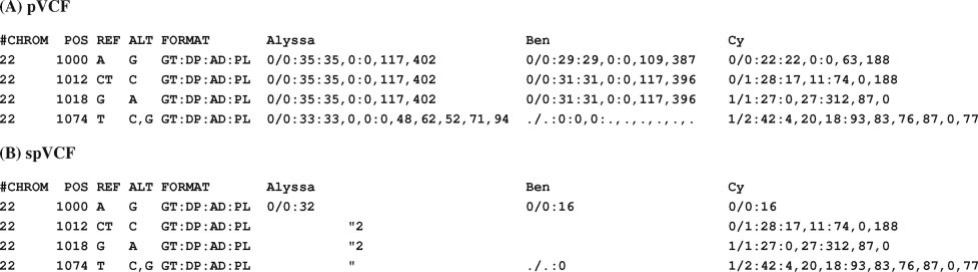
spVCF encoding example. (**A**) Illustrative pVCF of four variant loci in three sequenced study participants, with matrix entries encoding called genotypes and several numeric QC measures. Some required VCF fields are omitted for brevity. (**B**) spVCF encoding of the same example. QC values for reference-identical and non-called cells are reduced to a power-of-two lower bound on read depth DP. Runs of identical entries down columns are abbreviated using quotation marks, then runs of these marks across rows are length-encoded. Cy’s entries are shown column-aligned for clarity; the encoded text matrix is ragged

We sought an incremental solution to these challenges for existing pVCF-based pipelines, which may be reluctant to adopt fundamentally different formats or data models (Danek and Deorowicz, 2018; Deorowicz and Danek, 2019; Lan *et al.*, 2020; Layer *et al.*, 2015; Li, 2016; Zheng *et al.*, 2017; Supplementary Appendix S1) to minimize disruption to existing processes and users. To this end, we developed Sparse Project VCF (spVCF), which adds three simple features to VCF (**Fig. 1**):

1. *Squeezing: judiciously reducing QC entropy*. In those cells with zero reads supporting a variant (typically Allele Depth AD=d,0 for any *d*) and corresponding non-variant genotype, we discard all fields except the genotype GT and the read depth DP, which we also round down to a power of two (0, 1, 2, 4, 8, 16,…; configurable). Any cell reporting evidence of variation retains its original QC measures and other annotations.

This convention, inspired by common base quality score compression techniques, aims to preserve nearly all *useful* information, removing minor fluctuations in non-variant cells. (If required for compatibility, non-variant genotype likelihoods could be approximated from depth, albeit without read quality inputs that might subtly affect downstream calculations.)

1. *Succinct, lossless encoding for runs of reference-identical cells*. First, we replace the contents of a reference-identical (or non-called) cell with a double-quotation mark if it’s identical to the cell above it, compressing runs down the column for each sample. Then we run-length encode these quotation marks across the rows, so for example a stretch of 42 marks across a row is written <tab> "42 instead of repeating <tab>". The second, horizontal run-encoding step has negligible effect on zipped size, but should enable faster downstream processing, e.g. sample subset extraction. The QC squeezing synergizes with the run-encoding, by converting minor fluctuations into identical runs down each column.
2. *Checkpointing to facilitate random access* by genome range (row). While all *variant* genotype cells are readily accessible from a given spVCF row, fully decoding the remaining cells could require information from an arbitrary number of prior rows. Instead, the spVCF encoder periodically skips run-encoding a row, emitting a row identical to the squeezed pVCF. Each run-encoded row indicates the position of the last such *checkpoint* row, from which decoding can commence.

Our Apache-licensed Unix tool spvcf provides subcommands to (i) squeeze and run-encode pVCF to spVCF, (ii) squeeze pVCF without run-encoding (producing valid pVCF usually much smaller, albeit not as small as spVCF) or (iii) decode spVCF back to pVCF. If a spVCF file is compressed using bgzip, then tabix can create an index for it (Li, 2011) based on the unchanged locus-level VCF fields. A subcommand of spvcf used instead of tabix can then access the file by genome position, generating a standalone spVCF slice.

## 3 spVCF for DiscovEHR and UK Biobank

We tested spVCF on two large WES studies based on different upstream variant-calling pipelines.

First, using N=50 000 WES from the DiscovEHR study (Dewey *et al.*, 2016), we reduced a GATK-based pVCF file with 620 782 chromosome 2 variant loci from 79GiB vcf.gz to a 5.2GiB spvcf.gz file, 15× size reduction. Most of this reduction (6.9×) was achieved by the QC squeezing, while the run-encoding contributed 2.2×. Experiments with nested subsets of these N=50 000 WES indicate spvcf.gz file sizes growing roughly with $N^{1.1}$, compared to the original’s $N^{1.5}$ (Supplementary Fig. S1). VCF’s binary equivalent, BCF, reduces this example by 1.2× losslessly and exhibits the same $N^{1.5}$ scaling.

Second, with N=302 342 WES from UK Biobank (Van Hout *et al.*, 2020), spVCF reduced vcf.gz files for 252 610 loci in ten representative chromosome 2 segments from 110 to 7.7 GiB (Supplementary Table S1). This 14× combined ratio is similar to that achieved for DiscovEHR; decomposed however, QC squeezing was relatively less impactful (4.2×) and run-encoding relatively moreso (3.4×). On the one hand, the UK Biobank pVCF files were produced using a different upstream pipeline (‘SPB’) that already omitted genotype likelihoods for most reference-identical cells, leaving less to be squeezed out compared to DiscovEHR. On the other hand, the run-encoding’s effectiveness improved along with the 3.3×-higher variant locus density in the larger cohort, a trend expected to continue with larger *N*.

In single-threaded tests (Supplementary Appendix S2), spvcf encoded raw pVCF slightly faster than bgzip compresses it (both tools also have multithread modes). The decoder, with inputs and outputs both much smaller than the original pVCF, is several times faster. This makes it feasible to store spVCF files and decode them to pVCF only for transient use when needed.

## 4 Discussion

spVCF is practical ‘next step’ for storage and transfer in ongoing cohort sequencing projects, delivering far-reduced size growth and performant interoperability with existing pipelines. Upstream, joint-calling tools can stream their output pVCF into spvcf for now, and perhaps eventually generate spVCF natively. Downstream, population analysis tools can stream decoded pVCF from spvcf, with the future possibility of consuming spVCF directly.

spVCF clears a path to scale up the VCF data model to N≥1M WGS studies, notwithstanding residual super-linear size growth likely due to multiallelic loci and depth fluctuations. Meanwhile, many investigators—pacing with new sequencing technologies—are developing haplotype-centric paradigms that might eventually replace VCF. 

*Financial Support*: XB, WJS, and JGR are employees of Regeneron Pharmaceuticals, Inc.

*Conflict of Interest*: none declared.

## Supplementary Material

btaa1004_Supplementary_Data
