# Supplementary material for: Sparse Project VCF: efficient encoding of population genotype matrices
Source: Bioinformatics. 2020 Dec 10;36(22-23):5537–8. doi: 10.1093/bioinformatics/btaa1004 (PMC8016461; doi:10.1093/bioinformatics/btaa1004)
Supplement: btaa1004_Supplementary_Data [file btaa1004_supplementary_data.zip › spVCF_supplement_revised.pdf]

SUPPLEMENTARY DATA

Sparse Project VCF: efficient encoding of population genotype matrices

Appendix 1: Comparing spVCF to other VCF alternatives. spVCF's selective entropy reduction (QC squeezing), on top of lossless compression, complicates mutually-fair benchmarks with published pVCF-alternative formats (see main text references). GQT, bgt, GTC, and GTShark compress genotypes but aren't intended to store the sequencing QC measures, which are typically larger to begin with, and their omission is suitable for only some use cases. BCF (benchmarked on DiscovEHR below) and SeqArray can store all QC measures losslessly, with better compression than vcf.gz, but far short of the >tenfold improvements & flattened scaling in N reported here (with squeezing). genozip is mainly lossless but also has an optional entropy reduction for certain numerical fields, which is much less aggressive than spVCF's; its unspecified binary file format and more-restrictive licensing also stand in contrast.

We also note related approaches that have been open-sourced but not yet formally published; SAV[1] and Tachyon[2], which can be considered in the above categories, and a new format for the gnomAD and Hail projects[3], developed concurrently with spVCF and focused slightly upstream by extending the "gVCF" data model.

[1] https://github.com/statgen/savvy/blob/d11d790/sav_spec.md
[2] https://github.com/mklarqvist/tachyon
[3] https://gnomad.broadinstitute.org/blog/2019-10-gnomad-v3-0/

Appendix 2: 1000 Genomes Project WGS. We also applied spVCF to the N=2,504 1000 Genomes Project phase 3 WGS samples (doi:10.1038/nature15393), recently resequenced at New York Genome Center[4]. The draft GATK joint call set[5] totaling 1,250 GiB vcf.gz were reduced 5X to 250 GiB spvcf.gz. In this case, almost all (90%) of the reduction results from QC squeezing. The run-encoding has minimal effect on the short runs of similar read depths observed with the wider spacing of variant loci in the smaller N=2,504 cohort (compared to DiscovEHR N=50K and UK Biobank N=300K). While this application exhibits only a fraction of spVCF's potential, it has the advantage of fully public data access. Further information:

https://github.com/mlin/spVCF/blob/master/doc/1000G_NYGC_GATK.md

The new 1000 Genomes VCF files can be used to verify that `spvcf encode` is "slightly faster" than `bgzip -c`. This comparison between tools with different functions is meant to indicate that introducing spVCF into existing pipelines is unlikely to cause significant speed or cost bottlenecks. Using these invocations with the 129 GiB uncompressed VCF for chromosome 22 (originally 19GiB compressed),

```
pv -apbe CCDG_13607_B01_GRM_WGS_2019-02-19_chr21.recalibrated_variants.vcf \
    | spvcf encode > /dev/null
pv -apbe CCDG_13607_B01_GRM_WGS_2019-02-19_chr21.recalibrated_variants.vcf \
    | bgzip -c > /dev/null
```

Taking each tool's fastest of five such trials, `spvcf` completed this in 1,008s compared to 1,925s for `bgzip`. These tests used `spvcf` v1.1.0 and `bgzip` v1.10.2-3 (with libdeflate), each single-threaded on Intel i7-8650U CPU @ 1.90GHz; both tools also have multi-thread modes.

Acknowledgements. We are grateful to the Global Alliance for Genomics and Health, Large-Scale Genomics Work Stream for motivating discussions and early feedback on this work; particularly Albert Smith, Yossi Farjoun, Louis Bergelson, Chris Vittal, Cotton Seed, Cristina Gonzalez, Petr Danecek, Marcus Klarqvist, Rishi Nag, Richard Durbin, Thomas Keane, and Ewan Birney.

---

[4] https://www.internationalgenome.org/data-portal/data-collection/30x-grch38

[5] http://ftp.1000genomes.ebi.ac.uk/vol1/ftp/data_collections/1000G_2504_high_coverage/working/20190425_NYGC_GATK/

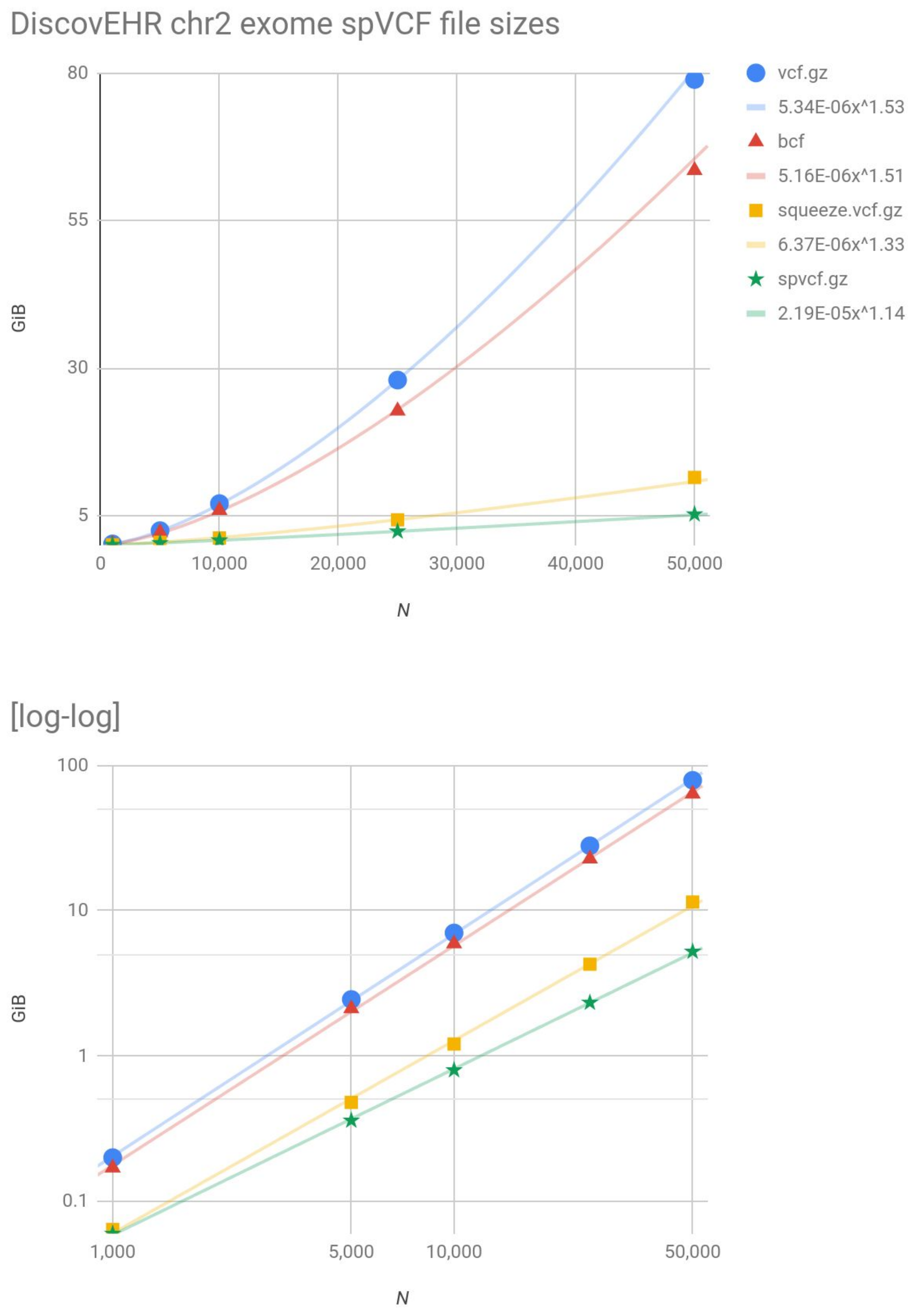

Figure S1. pVCF file sizes for DiscovEHR variants on chromosome 2 WES targets, generated with a GATK-based pipeline (nested subsets from N=1K to N=50K), with different formats and encodings: full-size pVCF with bgzip compression (vcf.gz), the BCF lossless binary equivalent (bcf), pVCF with QC squeezing but *not* run-encoding (squeeze.vcf.gz), spVCF with both squeezing and checkpointed run-encoding (spvcf.gz). All bgzip invocations used its default compression settings.

UK Biobank WES file sizes

chr2 segment	vcf.gz (MiB)	squeeze.vcf.gz (MiB)	spvcf.gz (MiB)	vcf:squeeze ratio	squeeze:spvcf ratio	vcf:spvcf ratio
2_73280544_74448466	**12,052**	**2,581**	**625**	**4.7**	**4.1**	**19.3**
2_189057928_191355427	**11,178**	**2,807**	**857**	**4.0**	**3.3**	**13.1**
2_217854536_218745461	**11,743**	**2,655**	**722**	**4.4**	**3.7**	**16.3**
2_200894336_202119329	**11,292**	**2,745**	**826**	**4.1**	**3.3**	**13.7**
2_113639764_120153584	**11,201**	**2,809**	**887**	**4.0**	**3.2**	**12.6**
2_178768414_182957024	**11,194**	**2,775**	**835**	**4.0**	**3.3**	**13.4**
2_208381175_213972047	**11,191**	**2,717**	**783**	**4.1**	**3.5**	**14.3**
2_182957025_189057927	**11,099**	**2,801**	**895**	**4.0**	**3.1**	**12.4**
2_25150828_26567400	**11,478**	**2,671**	**766**	**4.3**	**3.5**	**15.0**
2_241226877_242193529	**10,551**	**2,419**	**673**	**4.4**	**3.6**	**15.7**

Table S1. pVCF file sizes and compression ratios for *N*=302,342 UK Biobank WES, showing ten representative chromosome 2 segments covering 252,610 loci in WES target regions. The breakdown by segment illustrates some variability within consistent overall trends (average vcf:spvcf ratio 14.6±2.1). The UK Biobank data are generated with a non-GATK pipeline ("SPB"; van Hout et *al.* 2019) illustrating spVCF's interoperability.
